# In an East Borneo Hospital.—II

**Published:** 1912-09-21

**Authors:** 


### IN AN EAST BORNEO HOSPITAL.—II.

(BY OUR SPECIAL COMMISSIONER.)

Walking round the wards with Dr. Horst, the first thing that struck me was the fact that here, in this new hospital of the Petroleum Company, the cost per bed must be manifestly much lower than it is in any European establishment of a similar kind. To begin with, the patients need no bed clothes—in fact, they resent them. The Malay sleeps on his mat, which is spread over the wire mattress of the iron bedstead. He does not even want a pillow. The Chinese patients demand their own pillows in the shape of hard wooden blocks that have a deep concavity for the neck, resembling very much the block used during the Terror on the guillotine. It seems a most uncomfortable pillow, but they like it and want it. Their food, which is admirably cooked in the fine kitchen attached to the institution, is cheap, for it consists largely of rice and fish. Beri-beri patients get more substantial fare, and as a prophylactic beans are served out instead of rice.

### The Patients in the Wards.

In the first ward we enter are some forty coolies. The beds are ranged round the walls, and the ward is more crowded than it ought to be, since there are more beds than the floor space warrants. There are all kinds of diseases here and all types of patients. Everyone who enters is examined, as a matter of routine, for the presence of ankylostoma, a parasite that is responsible for a vast deal of the anæmia so commonly observed in both natives and Europeans in Insulinde. If ova are found a course of eucalyptol, followed and preceded by the useful dose of salts, is prescribed as the routine treatment. It is usually sufficient to effect a cure but relapses are frequent. Here is a sufferer from beri-beri, a disease that at one time caused frightful ravages in the army in Atjeh, but which now is found to yield well to treatment. In this patient the outlook is bad. He is swollen round the neck, the parotid and submaxillary glands are massively enlarged, and there is considerable œdema. "These cases," remarks Dr. Horst, "are very dangerous. The great risk is sudden heart failure, of which the books make almost no mention." Percussion reveals the fact that this patient's cardiac dulness is already much increased. It is a real dilatation, not, as so commonly stated, a hydro-pericardium. Death ensues suddenly and unexpectedly as the patient is lying down, so that these cases need constant watching.

On the next bed is an emaciated patient who, beyond his extreme degree of thinness, his contracted pupils, and his quickened pulse-rate, presents no signs. "He is an opium smoker," says the Doctor, "who comes to try and get cured of the habit. We are only too glad to help them by keeping them in hospital and prescribing good food and tonic treatment." The next patient has come in for fever. He has swollen knees and complains of pain in the ankle, but he has had no definite rigor. The type of fever here often presents these symptoms, sometimes in such a form that it gives the picture of an attack of Malta fever. Examination, however, reveals the carefully concealed fact that the man is suffering from gonorrhœal arthritis. Here, again, is a convalescing patient who has had his left foot amputated by a Pirogoff owing to a severe crush. The wound has healed remarkably well, and he now walks about the ward in a way that proves the excellence of the method. In a side ward, which is private, we find a woman who has been under treatment for exophthalmic goitre with severe cardiac signs. She is almost well and wants to go home, but is persuaded to stay a few days longer. There are few female nurses and the presence of a well-trained European nurse would be a great help. Probably the institution will have the aid of one or two in the near future, for at Batavia there are several excellent nurses and the training schools in Holland are fully alive to the need in Insulinde.

### The Infectious Diseases Ward.

Now we enter the ward reserved for infectious diseases, which is situated at the other extremity of the hospital encampment. It is in itself a remarkable ward, for it is isolated by a high barbed-wire fence, and its doors, windows, and various outlets are guarded by mosquito-proof netting. Overalls and rubber overshoes are provided for every attendant who enters here. The need for the rubber shoes is apparent the moment one enters. The dysentery patients do not scruple to perform their necessary evacuations on the floor, for the need is usually so great that they have no time, even if they have the strength, to rush to the adjacent lavatories. The nursing staff of this ward is small and cannot cope with the patients in the manner that would be followed in a European hospital, so that, although bed pans are used, the floor of the ward has constantly to be flushed with the strong creolin solution which is kept ready for that purpose. Nevertheless, the interior of the ward presents a clean appearance and the atmosphere is quite sweet. This is only obtained by strict attention to the flushing which has to be done immediately.

Dysentery is here one of the hardest and worst diseases to cope with. The ipecacuanha treatment has quite failed to realise the high expectations held out; now the favourite remedy is small doses of bismuth and opium frequently repeated. This serves well for the amœbic cases. For the bacteriological form of the disease, which is much more rapid and dangerous, the best, and, indeed, the only remedy, appears to be the serum treatment which is actively carried out. Cholera is fortunately unknown, except in rare instances—a great contrast to the hospitals in Java and Sumatra, where this disease is by no means uncommon. Plague is equally conspicuous by its absence. On the other hand, choleraic diarrhœa is frequent, and smallpox, beri-beri, and sometimes dengue, demand the utmost precaution in the matter of treatment and isolation.

On leaving the ward the visitor is invited to wash in creolin solution and to immerse his boots literally in a much stronger solution before he pulls them off. Only by observing these precautions can one be sure of not conveying infection.

### The Staff and Native Assistants.

A visit to the other wards, which are arranged on the same plan, makes the stranger acquainted with the many skin diseases which are prevalent here, the majority caused through parasites, though a good many are syphilitic lesions. I inspected the bathing and lavatory accommodation provided. This is very good in nearly every case. The w.c.s are well looked after, and the big water tanks that serve the douches are regularly emptied and provided with mosquito-proof covers, for they afford excellent breeding-places to these troublesome insects. Although it is not proved that fever here is carried by the mosquito, the probabilities are that it is, and so every possible precaution is taken. The fens and marshes on the outskirts of the town have been liberally saturated with petroleum, and the number of the insects has sensibly diminished during the last few years.

While the Balik Papan hospital cannot claim to be up to date from the point of view of the European hospital superintendent, it is a most interesting institution, and when one considers that it is in charge of a single medical officer, who has a large private practice to look after, and who is only helped by native assistants whom he has had to train himself from the very beginning, one stands astonished at the work that it does so successfully. I came away with the firm conviction that one of the most valuable assets that Balik Papan possesses is Dr. Horst himself. He is a keen scientist, a most sympathetic attendant, and a first-class manager. For such a man to find time to do research work and to keep abreast of the recent advances in treatment, bacteriological and serum diagnosis in the manner that he does is marvellous, and offers a striking contrast to the medical superintendents of our own infirmaries who imagine that they have enough to do in managing their institutions, and therefore find no time to avail themselves of the fine material waiting for exploitation at their hands. I have necessarily only been able to give a brief and cursory epitome of the many aspects that the hospital presents. To go fully into details of structure, management, and treatment would need far more space than is at my disposal. All I can say is that no one interested in hospitals who visits the Dutch East Indies should omit to pay a visit to Balik Papan and its excellent hospital, where some six hundred patients can be accommodated, and where half that number is under daily treatment.

